# Bioaccumulation and Biotransformation of Chlorinated Paraffins

**DOI:** 10.3390/toxics10120778

**Published:** 2022-12-12

**Authors:** Liujun Chen, Bixian Mai, Xiaojun Luo

**Affiliations:** 1 State Key Laboratory of Organic Geochemistry and Guangdong Key Laboratory of Environmental Resources Utilization and Protection, Guangzhou Institute of Geochemistry, Chinese Academy of Sciences, Guangzhou 510640, China; 2 University of Chinese Academy of Sciences, Beijing 100049, China; 3 Guangdong-Hong Kong-Macao Joint Laboratory for Environmental Pollution and Control, Guangzhou Institute of Geochemistry, Chinese Academy of Sciences, Guangzhou 510640, China

**Keywords:** chlorinated paraffin, occurrence, bioaccumulation, biotransformation, distribution, metabolism

## Abstract

Chlorinated paraffins (CPs), a class of persistent, toxic, and bioaccumulated compounds, have received increasing attention for their environmental occurrence and ecological and human health risks worldwide in the past decades. Understanding the environmental behavior and fate of CPs faces a huge challenge owing to the extremely complex CP congeners. Consequently, the aims of the present study are to summarize and integrate the bioaccumulation and biotransformation of CPs, including the occurrence of CPs in biota, tissue distribution, biomagnification, and trophic transfer, and biotransformation of CPs in plants, invertebrates, and vertebrates in detail. Biota samples collected in China showed higher CP concentrations than other regions, which is consistent with their huge production and usage. The lipid content is the major factor that determines the physical burden of CPs in tissues or organs. Regarding the bioaccumulation of CPs and their influence factors, inconsistent results were obtained. Biotransformation is an important reason for this variable. Some CP congeners are readily biodegradable in plants, animals, and microorganisms. Hydroxylation, dechlorination, chlorine rearrangement, and carbon chain decomposition are potential biotransformation pathways for the CP congeners. Knowledge of the influence of chain length, chlorination degree, constitution, and stereochemistry on the tissue distribution, bioaccumulation, and biotransformation is still scarce.

## 1. Introduction

Chlorinated paraffins (CPs), known as poly-chlorinated n-alkanes (PCAs), are produced by the chlorination of different n-alkanes with the formula C_n_H_2n+2−m_Cl_m_. They are divided into short-chain (SCCPs, C_10–13_), medium-chain (MCCPs, C_14–17_), and long-chain CPs (LCCPs, C ≥ 18) according to the lengths of their carbon chain. Recently, very-short-chain CPs (vSCCPs, chain lengths below 10) have been reported in wildlife and human samples [1,2]. CPs have the advantages of low volatility, flame retardancy, good electrical insulation, and low cost. Consequently, CPs have been used as plasticizers, flame retardants, and additives in metal cutting fluids, adhesives, coatings, rubber, and sealants with a global CP production of more than two million tons per year [3,4].

CPs are highly persistent in the environment, are bioaccumulative and toxic to animals and humans, and have a high probability of undergoing long-range environmental transport. SCCPs were designated as persistent organic pollutants (POPs) and listed in annex A of the UN Stockholm Convention in 2017. This regulation has resulted in a limit in the use and production of SCCPs. A substantial amount of research has reported the occurrence of CPs in abiotic media such as sediment [5], soils [6], and airs [7], as well as in the biota [8]. Nevertheless, the understanding of the environmental behavior, fate, and potential risks is still scarce because of the extremely complex CP congeners and the difficulties regarding accurate quantification.

Some reviews on CPs have been produced in the last ten years, and most of them focused on analysis method, environmental distribution, and toxicity [9,10,11,12]. The data on CPs in organisms have increased over the last decade, and studies on the trophic transfer of CPs in both aquatic and terrestrial food webs has increased substantially in recent years [13,14]. The concerns regarding the biotransformation of CPs have also been continuously increasing. Thus, the aim of the present study is to compile our present knowledge on the bioaccumulation and biotransformation of CPs in biota and identify knowledge gaps, i.e., to some further research needs. The review is based on data retrieved from studies primarily on chlorinated paraffins in biology (published between 1982 and 2022). We searched the databases Web of Science and CNKI as well as websites from national and international organizations such as the U.S. EPA, the World Health Organization (WHO), and the EU. The search strategy was based on the following keywords: chlorinated paraffin, occurrence, tissue distribution, bioaccumulation, biotransformation, and metabolism. We consulted 627 references in total, and based on the objectives of this review, 115 were selected and classified into 5 major categories: (i) Occurrence of CPs in organisms, including aquatic biota, terrestrial biota, humans and human foodstuffs samples (68 references); (ii) Tissue distribution of CPs in organisms (9 references); (iii) Bioaccumulation of CPs in organisms (25 references); (iv) Biotransformation of CPs in organisms, including plants, invertebrates, and vertebrates (22 references).

## 2. Occurrence of CPs in Organisms

Data on CPs in biota were mostly obtained from China from 2010 onwards (Table 1). This is plausible considering that China has been the largest global producer and consumer of CPs since 2007, producing up to 1,000,000 metric tons in 2009 [15], and CP contamination has become a major environmental concern in China. A precise comparison of CP concentrations among different regions or countries is difficult since the samples were collected in different periods and the species differed between different studies. Additionally, the concentrations were expressed using different units (ng/g dw, ng/d ww, ng/d lw, etc.). Generally, CP concentrations in aquatic organisms from China were several orders of magnitude higher than those from other regions or countries (Table 1). Relatively high CP concentrations were found in aquatic organisms collected from e-waste recycling sites such as those in Qingyuan and Taizhou, China [13,16,17]. SCCPs have also been reported at relatively high concentrations in mollusks from the Liaohe estuary [18] and fish from Liaodong Bay [19] (Table 1).

Data on CPs in terrestrial biota are all from China. Similar to aquatic organisms, high SCCP concentrations were also detected in terrestrial biota collected from e-waste recycling areas [16]. Surprisingly, CP concentrations in plants and animals collected from Tibet, China, were also comparable with those in the e-waste recycling area [14,51,52], indicating high CP pollution in this area, although the intensity of anthropogenic activities in this area was thought to be lower.

SCCPs and MCCPs have been detected in human tissues including fingernails, hair, blood, cord serum, the placenta, and breast milk from different countries [55,56,57,58,59,60,61,62,63,64,65,79,80]. Human foodstuffs, including aquatic food, meat, baby food, noodles, green tea, cereals and beans, milk, rice seeds, and oil food, from China and Europe were also found to contain SCCPs and MCCPs [46,66,67,68,69,70,71,72,73,74,75,76,77,78]. Concentrations of SCCPs in the foodstuffs were generally higher than concentrations of MCCPs, with the exception of oil-based vitamin E dietary supplements collected in Germany [78]. However, the concentrations of MCCPs were similar to or more than the concentrations of SCCPs in most of the human tissues samples, indicating a higher bioaccumulation of MCCPs than SCCPs in humans.

Earlier studies focused more on SCCPs in aquatic species; however, research on vSCCP, MCCPs, and LCCPs in terrestrial species or humans has increased in recent years [2,22,44]. LCCPs were first detected in human blood samples in China with median concentrations of 150 ng/g lw, which were lower than those of SCCP (3500 ng/g dw) and MCCPs (740 ng/g dw) [61]. Since then, LCCPs were detected in marine organisms from the Baltic Sea (nd: 130 ng/g dw) [34], Greenland and Iceland (<0.41–930 ng/g dw) [1], aquatic and terrestrial organisms from Germany (nd: 2400 ng/g dw) [44], plants from Beijing, China (27–561 ng/d dw) [54], and cereal and legume samples from China (48.1–664 ng/g dw) [59]. Compared with SCCPs and MCCPs, the concentrations of LCCPs in the abovementioned samples were on the same order of magnitude as those of SCCPs and MCCPs. vSCCPS were initially quantified in aquatic biota samples from the Yangtze River Delta in China with high levels (2.6–8400 ng/g lw) [2] compared to those of vSCCPs in bivalves and marine mammals from Greenland and Iceland (<0.12–34 ng/g lw) [42] and those in biota from Germany (nd: 65 ng/g lw) [44]. vSCCPs were also detected in fish and mosses from Liaodong Bay and the Antarctic at relatively low levels (3.4–153 ng/g dw) [22].

## 3. Tissue Distribution of CPs in Organisms

Several in vivo exposure experiments investigated the tissue distribution of CPs in organisms [81,82,83]. Lipid content was the main factor in determining the physical burden of CPs in organ tissue. The high concentrations of SCCPs (based on wet weight) were in the abdominal fat and the feces, and the low concentrations were in the blood, meat, and bile fluid when broiler chickens were exposed to SCCP (60% Cl) via diet. An exposure experiment using laying hens obtained similar results [82]. The amount of SCCP deposited in the leg meat was higher than that in breast meat due to the higher fat content of leg meat compared to breast meat in both broiler and laying chickens. An experiment using rat exposure to SCCPs, MCCPs, and LCCPs revealed that approximately 57.0–76.5% of CPs were deposited in the liver, 23.1–42.4% of CPs in the fat, while approximately 0.3–1.4%, 0.01–0.04%, and 0.002–0.022% CPs were in the blood, kidneys, and lungs, respectively [83]. The same dependence on the lipid content was also observed in field fish samples. Sun et al. (2017) [13]. found concentrations of SCCPs (based on wet weight) in snakehead and mud carp in the order liver > gill > kidney > skin > muscle, and the SCCP concentrations in the tissue were positively correlated with the content of lipids in tissues (*p* < 0.001). However, when the concentration was expressed based on lipid, the muscle SCCP concentrations were the highest among all tissues in the laying chicken [84]. Sun et al. (2020) found similar results in chicken collected from an e-waste recycling site [49]. The concentrations of SCCPs were in the order muscle (6200 ng/g lw) > fat (2400 ng/g lw) > liver (1100 ng/g lw). This tissue distribution was due to the low lipid content in muscle and metabolism of SCCPs in the liver.

The deposition of CPs in tissue was also related to carbon chain and degree of chlorination. Mézière et al. (2021) [84] conducted an experiment using laying hens exposed to different degrees of chlorination (low %Cl and high %Cl) SCCPs, MCCPs, and LCCPs to investigate the accumulation and distribution of CPs in biota after ingestion. All C_10_–C_36_ CPs were detected in the liver. However, differences were observed in CP distribution: LCCPs with high %Cl were retained in the liver, while LCCPs with low %Cl circulated through the serum and were distributed in the different compartments but were mostly excreted through the eggs; SCCPs and MCCPs were found in all tissues at similar levels. SCCPs with low %Cl were detected at lower levels compared to SCCPs with high %Cl and MCCP, implying a higher biodegradation potential for SCCPs with low %Cl compared to CPs with higher %Cl. Du et al. (2020) [85] analyzed the vSCCPs, SCCPs, MCCPs, and LCCPs in different tissues of terrestrial short-tailed mamushi (*Gloydius brevicaudus*) and the semiaquatic red-backed rat snake (*Elaphe rufodorsata*) from the Yangtze River Delta of China. The tissue distribution of total CPs content (ww) in the two snakes was in the order fat (44–1300 ng/g ww) > muscle (39–550 ng/g ww) > liver (41–490 ng/g ww). vSCCPs (C6–9) and SCCPs (C10–13) were preferentially distributed in the snake liver, while fat was an important storage compartment for MCCPs (C14–17) and LCCPs (C > 18).

The tissue distribution of CPs also exhibited the specific homologues for specific tissues. In the studies of CPs in chicken, Sun et al. (2020) [49] observed that the concentration ratio of muscle to liver of CPs decreased with increased log *K*_OW_, while the concentration ratio of fat to liver increased with increasing log *K*_OW_. This result indicated that the fat prefers to accumulate high log *K*_OW_ homologues compared to the liver and muscle. Muscles that preferred to deposit SCCP with lower log *K*_OW_ compared to liver were also found in fish samples [13]. The difference in the lipid component was responsible for this tissue distribution. The proportions of neutral lipids (triglycerides) to total lipids in the abdominal adipose tissue, liver, and muscle of chickens were 98%, 52%, and 32%, respectively, while the proportions of polar lipids (phospholipids) to lipids were 1.7%, 46%, and 65%, respectively [63,86]. It is likely that SCCPs with higher log *K*_OW_ have a stronger affinity with neutral lipids than polar lipids.

## 4. Bioaccumulation of CPs in Organisms

Bioaccumulation is the process of accumulation of chemicals in an organism from the environment through diet, skin absorption, or respiration that occurs when the chemical concentration in the organism exceeds that of the surrounding environment or diet after reaching equilibrium [87]. The bioaccumulation potential of chemicals for evaluation comprises the bioaccumulation factor (BAF), bioconcentration factor (BCF), biomagnification factor (BMF), and trophic magnification factor (TMF). Generally, the chemical is considered to be bioaccumulative when BAF/BCF > 5000, BMF/TMF > 1 [88].

Numerous laboratory and field studies showed that CPs had bioaccumulation potential. The values for the logBAF of ∑SCCPs and ∑MCCPs were estimated as 2.70–4.45 and 2.25–3.64, respectively, for aquatic insects from Qingyuan at an e-waste recycling site [20], 1.48 for soil–vegetation of ∑SCCPs from the Arctic [35], 4.5–6.05 for fish of ∑SCCPs from Liaodong Bay [19,23], and 2.04–3.79 for marine organisms of ∑SCCPs from the East China Sea [27]. Following the administration of ^14^C-labeled CPs in bivalve blue mussels (*Mytilus edulis*), the values for the BAF of MCCPs (C_16_, 34% Cl) and SCCPs (C_12_, 69% Cl) were estimated at 7000 and 13,900, respectively, indicating that SCCPs with short carbon chains and high chlorination had strong bioaccumulation [89]. The log BCF values of five different CPs (Cereclor S45:MCCP 45% Cl; Cereclor 50LV: SCCP 50% Cl; Huels 70C:SCCP 70% Cl; CP-42: 42% Cl, C10–C17, C21–C31; CP-52: 52% Cl; C_9_–C_29_) in Daphnia magna were assessed as 6.7–7.0 (Lkg lipid^−1^). All the CPs tested were bioaccumulative in D. magna [90].

The BMF of ∑SCCPs for oyster–mangrove crab (2.4) from the Pearl River Estuary [25], Ngas–Agas (1.9) from Antarctica [35], and prey species–finless porpoises (3.1–6.7) and prey species–Indo-Pacific humpback dolphins (11–38) from Hong Kong [21] were all >1, indicating the biomagnification of SCCPs in these marine food chains. In juvenile rainbow trout that were exposed to CPs (C_10_H_15_._3_Cl_6_._7_, C_14_H_23_._3_Cl_6_._7_ and C_18_H_31_._4_Cl_6_._6_), the BMFs for ∑SCCPs ranged from 0.9 to 2.8, demonstrating that these CPs had the potential to biomagnify [91]. In an e-waste recycling pond in South China, the BMF values for ∑SCCPs and ∑MCCPs for fish–water snakes were 2.9 and 2.95, respectively, indicating biomagnification [17]. The mean BMFs of vSCCPs, SCCPs, MCCPs, and LCCPs in the food chain (black-spotted frog–red-backed rat snake) were 2.2, 1.9, 1.8, and 1.7, respectively, indicating the potential for biological magnification. This was the first study to address the biomagnification potential of vSCCPs and LCCPs [85]. On the other hand, biodilution was also reported in both marine and freshwater food chains. For examples, the BMFs of SCCPs and MCCPs for fish–bird in an e-waste recycling pond were 0.08 and 0.1, respectively [17]. The BMFs of ∑SCCP were 0.46 for gammarid–cod from Ny-Ålesund in the Arctic [36], and were 0.282 and 0.212, respectively, for plant–plateau pika, as well pika–eagle from the Tibetan plateau [14].

The varied trophodynamic behavior of CPs was also reported in previous studies. Trophic magnifications of SCCPs were observed in an aquatic food web from Dianshan Lake in Shanghai (TMF: 1.19–1.57) [30], marine food web from the East China Sea (TMF: 3.98) [27], and zooplankton–shrimp–fish food web (TMF: 2.38) [19] and fish food web (2.57) [23] from the Liaodong Bay Sea. The TMF of SCCPs and MCCPs in a marine food web from Hong Kong (TMF: 4.29 and 4.79, respectively) [21] and in a terrestrial food web dominated by insects from an e-waste recycling site in southern China (TMF: 2.08 and 2.45, respectively) indicated trophic magnification [13]. The TMF of SCCPs and MCCPs in the invertebrates–forage fish–lake trout food webs from Lake Michigan and Lake Ontario were 0.97 ± 0.33 and 1.2 ± 0.51, respectively, which is in the margin of trophic magnification and trophic dilution [92]. The lake trout had lower SCCP concentrations than several of their prey on a lipid basis, which did not support the biomagnification of CPs. Meanwhile, the trophic dilution of CPs in food webs was also reported. The TMF of SCCPs in an aquatic food web from an e-waste recycling site was 0.17 [13]; the TMF values of SCCPs and MCCPs in a freshwater food web from France were <1 [93]; the TMF of SCCPs in mollusks from Bohai was 0.396; and the TMF of SCCPs in plant–pike–eagle food chain from the Tibetan plateau was 0.392 [14].

The biomagnification and biodilution of CPs in both aquatic and terrestrial food webs were reported in different regions and in different food webs. The structure of food webs; size of organisms collected; food habits; biotransformation of SCCPs in organisms; treatment method for any outliers; and even environmental parameters such as water temperature, dissolved organic matter, and suspended particles may have all contributed to the differences obtained [19,94,95].

Both the chlorine content and the carbon chain length determine the physicochemical properties of CPs [96]. Therefore, both may affect the bioaccumulation capacity of CPs. In a laboratory exposure experiment, pumpkin seedlings were exposed to four kinds of SCCPs: 1,2,5,6,9,10-C_10_H_16_Cl_6_ (HexCD), 1,1,1,3,8,10,10,10-C_10_H_14_Cl_8_ (OctaCD), 1,1,1,3,10,11-C_11_H_18_Cl_6_ (HexCU), and 1,1,1,3,12,13- C_13_H_22_Cl_6_ (HexCT). The cumulative amounts of OctaCD, HexCT, HexCU, and HexCD in pumpkin were 59.9%, 40.5%, 33.4%, and 23.6% of the parent SCCP, respectively, showing that bioaccumulation increased with the carbon chain length and chlorination degree in plants [97]. The soil–vegetation BAFs of SCCP in the Arctic support this finding. BAFs increased with increasing content carbon and chlorine, and the number of carbon atoms was the primary factor regarding the bioaccumulation of SCCPs [36]. Fisk et al. [91] observed that the BMFs of CP congeners (C_10_Cl_6.7_, C_14_Cl_6.6_, and C_18_Cl_6.7_) in juvenile rainbow trout increase with increasing carbon chain length in a given chlorination content. The BMF of SCCP congeners in a food chain (tapertail anchovy–Bombay duck) collected in the Pearl River Estuary showed significant positive correlations with both number of carbon and chlorine atoms of SCCP congeners [26]. All these results indicate that both the length of carbon chain and the chlorine content affect biomagnification of CPs in organisms.

However, some field monitoring provided inconsistent results. The BMFs of SCCP congeners in the food chain Agas–Ngas from Antarctica decreased from 2.3 for C_10_ to 0.8 for C_13_ [35]. Houde et al. (2008) [92] also observed that BMFs in a food web from Lake Michigan decreased with the increasing carbon chain length of SCCP congeners. In aquatic organisms collected from a large sewage treatment plant in Beijing, the BMF of SCCP homologues exhibited significant correlation with chlorine content while no significant correlation was found between the BMF and carbon chain length [94]. The correlation between bioaccumulative potential and chlorine content but not between carbon chain length were also found in other food chains/webs, such as bivalves–sediments from Bohai Sea [24], and fish–water snake, fish–waterbird food [17], and insect-dominated terrestrial food webs in an e-waste site in South China [50]. These results imply that chlorination degree rather than carbon length affects the bioaccumulative potential of CP congeners.

The low bioaccumulative potential with low chlorine content of these CPs may be due to the metabolization of low-chlorine-content CPs, which were more easily compared to CPs with higher chlorine content. The carbon chain length has positive and negative effects on the bioaccumulation of CPs. On the one hand, the lipophilicity of CP congeners increases with the increasing carbon chain length, which is beneficial to bioaccumulation. On the other hand, the molecular sizes of CP congeners increase with the increasing carbon chain length, which reduces the bioaccumulative potential of CP congeners. This could explain the inconsistent results of the observed effects of carbon chain on the bioaccumulation of CP congeners.

## 5. Biotransformation of CPs in Organisms

The biodilution and trophic dilution of CPs in food chains/webs were reported in both aquatic and terrestrial ecosystems. These abnormal trophic transfer behaviors were highly suspected to be related to the biotransformation of CPs in high-trophic-level organisms. The biotransformation of CPs was investigated in the early 1980s [98]. More studies on biotransformation of CPs have been conducted recently since SCCPs were listed as emerging POPs.

### 5.1. Biotransformation of CPs in Plants

**Figure 1:**
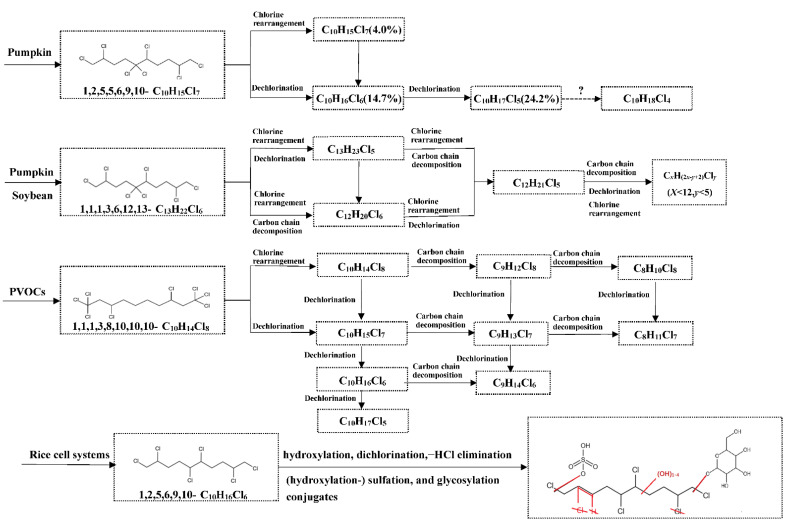
The transformation pathways of 1,2,5,5,6,9,10-C_10_H_15_Cl_7_, 1,1,1,3,6,12,13-C_13_H_22_Cl_6_, and 1,1,1,3,8,10,10,10-C_10_H_14_Cl_8_ mediated by pumpkin seedling, rice cell, soybean, and PVOCs, respectively. Values in the brackets are the transformation ratios of parent compound to the daughter compounds. The dotted arrow with the question mark is the pathway which possibly occurs but is not able to be detected by GC/ECNI-LRMS.

Li et al. [99] conducted a series studies on biotransformation of CPs in plants. 1,2,5,5,6,9,10-C_10_H_15_Cl_7_ was found to produce dechlorination products (C_10_H_17_Cl_5_ and C_10_H_16_Cl_6_ (14.7%) and chlorine rearrangement products (C_10_H_15_Cl_7_) in pumpkin plant (Figure 1). Further research on biotransformation of CPs (1,2,5,6,9,10-C_10_H_16_Cl_6_, HexCD; 1,1,1,3,8,10,10,10-C_10_H_14_Cl_8_, OctaCD; 1,1,1,3,10,11-C_11_H_18_Cl_6_, HexCU; 1,1,1,3,12,13-C_13_H_22_Cl_6_, HexCT) in pumpkin and soybean seedling indicated that dechlorination, chlorine rearrangement, and carbon chain decomposition products were detected (Figure 1). The biotransformation rate of SCCPs was higher in soybean than in pumpkin seedlings, which may be due to the higher fat content of soybean seedlings than pumpkin seedlings [97]. To verify that airborne chlorodecanes were converted by reactive phytogenic volatile organic compounds (PVOCs), the PVOCs of pumpkin collected in sealed glass bottle reacted with 1,1,1,3,8,10,10-C_10_H_14_Cl_8_ in a concentration of 96.4 ng/µL under illumination for 10 days, while the reaction control group did not contain PVOCs. Dechlorination, chlorine rearrangement, and carbon chain decomposition products were detected with or without PVOCs: C_10_Cl_5–8_, C_9_Cl_6–8_, and C_8_Cl_7–8_. The PVOCs of pumpkin seedlings promoted these transformations to a certain extent. In the absence of PVOCs, CPs may react with OH radicals in the atmosphere [100]

Chen et al. (2020) [101] investigated the biotransformation of CPs (1,2,5,6,9,10-C_10_H_16_Cl_6_ and 52% MCCPs) using suspension rice cell culture exposure systems. In total, 79.53% of 1,2,5,6,9,10-C_10_H_16_Cl_6_ and 40.70% of 52%-MCCP were metabolized by suspension rice cells, respectively. Forty and 25 metabolic products for 1,2,5,6,9,10-C_10_H_16_Cl_6_ and 52%-MCCP, respectively, were identified, including (multi-) hydroxylation, dechlorination, –HCl– elimination metabolites, (hydroxylation-) sulfation, and glycosylation conjugates. A comprehensive metabolic molecular network and potential degradation pathway were proposed. The results of the abovementioned study improved our understanding of the transformation behaviors of CPs in plant.

### 5.2. Biotransformation of CPs in Invertebrates

Degradation of CPs by microorganisms was firstly reported in the 1980s. Omori and Kodama (1987) [102] isolated four bacterial strains (HK-3, HK-6, HK-8, and HK-10) from soil. In the presence of n-hexadecane, cometabolic dechlorination of chlorinated paraffins was observed. The cometabolic dechlorination of CP-150 (C_15_._4_H_25_._4_Cl_5_._6_, 50% Cl) by the mixture of these four bacteria reached 51% in 36 h.

**Figure 2:**
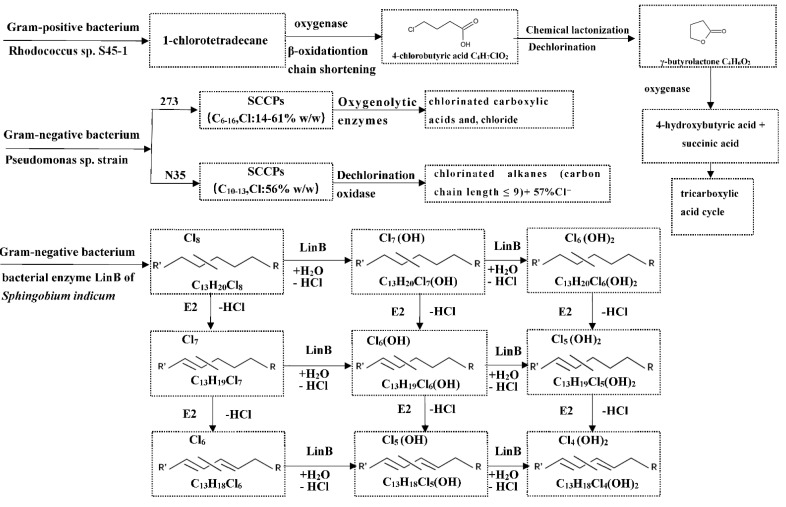
The transformation pathways of 1-chlorotetradecane, SCCPs (C6–16, Cl: 14–61% *w*/*w*), SCCPs (C10–13, Cl: 56% *w*/*w*), and octachlorotridecanes mediated by Rhodococcus sp. S45-1, Pseudomonas sp. Strain 273 and N35, and bacterial enzyme LinB of Sphingobium indicum, respectively. E2 are the reactions of abiotic and biotic elimination, which are not catalyzed by LinB.

Allpress and Gowland (1999) [103] isolated a bacterium, a species of Rhodococcus, later designated isolate S45-1, from a stream in Sheffield, UK. Isolate S45-1 was able to utilize chlorinated paraffins with chain lengths of between 10 and >20 as sole source of carbon and energy. The degradation product was identified as γ-butyrolactone (Figure 2). The pathway postulated for the formation of γ-butyrolactone involved initial attack at the nonhalogenated site by oxygenase, followed by chain shortening via β-oxidation, resulting in the formation of 4-chlorobutyric acid. Chemical lactonization of 4-chlorobutyric acid then leads to γ-butyrolactone formation, which is subsequently slowly catabolized to 4-hydroxybutyric acid and succinic acid, which allows entry to the tricarboxylic acid cycle. This is the first report of microbial utilization of chlorinated paraffins as sole source of carbon and energy. However, when the degree of chlorination was 58.5% (Cereclor S58) or greater, the growth of bacterium was inhibited.

In addition to Gram-positive bacteria, SCCPs were also degraded by Gram-negative bacteria, such as Pseudomonas. These bacteria were considered aerobic and abundantly distributed in soil, water, animals, and humans. Pseudomonas sp. strain 273 metabolized CPs with a good dechlorination effect, but similar to the strain screened by Allpress and Gowland (1999) [103], whose bacterial dechlorination cycle needed 20 days or even longer. Pseudomonas 273 strain could easily degrade monochlorinated alkanes whose chlorine atoms were located on primary carbons, and before β-oxidation, the chlorine atoms substituted at the end of SCCPs had to be dehalogenated enzymatically. However, the oxygenolytic dehalogenase enzymes produced by Pseudomonas strain 273 cannot metabolize SCCPs with chlorine atoms substituted in a vicinal arrangement [104].

Lu (2013) [105] isolated a bacterial strain identified as Pseudomonas sp. N35 from soil. This bacterial strain can utilize SCCPs as a sole source of carbon and energy. A total of 57.5% of chloride was released into the medium as chloride ions in pure culture within 20 days. Bioaugmentation resulted in 73.4% removal of SCCPs in the sludge microcosm after 30 days of treatment.

Heeb et al. (2019) [106] isolated several Sphingomonadacea strains which can convert hexachlorocyclohexanes (HCHs) from HCH dump site. The dehydrohalogenase expressed by such bacteria (also called as LinA2) can catalyze the HCl elimination reaction, which converts CPs to chlorinated olefins. However, not all SCCP congeners can be transformed by the bacterial enzyme LinA2. About 20–40% of the exposed CP material was reactive and readily converted by LinA2 within 24 h, while about 60–80% of the given CP material was not, or only slowly, converted by LinA2. In a subsequent study, Heeb and his colleagues [107,108] reported that under the catalysis of LinB enzyme, dehalohydroxylation reactions of CPs resulted in mono- and dihydroxylated products, and in mono- and diolefinic compounds that resulted in corresponding hydroxylated products. However, the LinB enzyme cannot catalyze HCl elimination to form alkenes. Conversion decreased with increasing degree of chlorination, while the effect of carbon chain length was less pronounced.

### 5.3. Biotransformation of CPs in Vertebrates

In the 1980s, studies of CPs metabolism were mostly performed with radioactive ^14^C-labeled CPs. Darnerud et al. (1982) [98] conducted a series of in vivo experiment to investigate the metabolism of CPs in animals. C57B1 mice were exposure to ^14^C-labeled chloroalkanes (i.e., C_12_H_16_._2_Cl_9_._8_, 68.5% chlorination; C_20_H_20_._1_Cl_5_._9_, 55.9% chlorination; and C_12_H_25_Cl_1_, 17.4% chlorination) via intravenous injection. After 12 h, 8%, 32%, and 52% of SCCP was exhaled through the respiratory system in the form of ^14^CO_2_, indicating that SCCPs were metabolized into metabolites in mice and CO_2_ was the final product. The exhaled amount of ^14^CO_2_ decreased with the increase of the degree of chlorination, implying that low-chlorination CP congener was readily to be metabolized. In the latter study, mice were transferred to an all-glass metabolism cage to monitor the exhaled air ^14^CO_2_ after the same administration above. After adding the cytochrome P-450 inhibitor piperonyl butoxide, the degradation rate decreased by 84%, which was positively correlated with the degree of chlorination, indicating that SCCPs with a high degree of chlorination were more dependent on cytochrome P-450 for degradation [109]. In carp, after intra-arterial (i.a.) injection of ^14^C-labeled chloroalkane (C_16_, 34% Cl), about 6% of dose was excreted as ^14^CO_2_ in 96 h. The metabolic rate in carp was lower than that of mice and quails (40% metabolized within 8 h) [110].

After gavage of ^14^C-labeled chloroalkane (C_16_H_30_._7_Cl_3_._3_, 34.1% Cl and C_12_H_20_._1_Cl_5_._9_, 55.9% Cl) to Japanese quail, radioactivity was highest in tissues with high metabolic activity and high cell turnover rate, and in bile and urine. About 40% and 20% of dose was excreted as ^14^CO_2_ in 8 h, respectively [111]. After oral and intravenous injection of ^14^C-labeled chloroalkane (C_16_H_20_._6_Cl_13_._4_, 69% Cl) to quail and C57B1 mice, about only 1% of dose was excreted as ^14^CO_2_ in 8 h. Obviously, the formation of ^14^CO_2_ was negatively correlated with the degree of chlorination of CPs [112].

Åhlman et al. (1986) [113] identified potential metabolites of polychlorinated hexadecane (^14^C-PCHD; 65% chlorine by wt) in rat. A uniformly ^14^C-labeled reagent was injected into the portal vein in bile-duct-cannulated rats (5–6 mg/kg) and the bile was collected for two or three days. The radioactivity was separated by ion-exchange chromatography into two major fractions: one acidic, the other amphoteric. N-acetylcysteine (mercapturic acid) and glutathione conjugation were found, which were produced by PCHD.

Dong et al. (2020) [83] reported the pharmacokinetic modeling for chlorinated paraffins in rats and humans via in vivo and in vitro exposed SCCP, MCCP, and LCCP to rat and liver microsomes. The metabolic rates of ∑SCCPs, ∑MCCPs, and ∑LCCPs were 1.31 × 10^−6^, 2.54 × 10^−6^, and 3.55 × 10^−6^/h in rat and 1.31 × 10^−6^, 2.54 × 10^−6^, and 3.55 × 10^−6^/h in humans. The metabolic rates of CPs were extremely slow and increased with the increased carbon chain length of CPs.

However, unlike the results of Dong et al. (2020) [83], an in vitro experiment using human liver microsomes conducted by He et al. (2021) [114] indicated that CPs were extensively and rapidly metabolized after incubation with human microsomes. After the incubation with CYP (Cytochrome P450) for two hours, a decrease of 85%, 98%, and 73% for SCCPs, MCCPs, and LCCP, relative to their negative controls, were observed. After Phase I and Phase II transformation, concentrations of different groups of CPs decreased by more than 70%, suggesting a rapid transformation of CPs by HLMs (human liver microsomes). The biotransformation products included vSCCPs, OH-CPs, CO-CPs, and COOH-CPs. Potential transformation pathway of CPs was C–C bone cleavage and ketones were potential products of biotransformation for CPs, especially for long-chain CPs (C > 17).

Lin et al. (2022) [115] reported the metabolism of SCCP in chicken and human via incubation of 1,2,5,5,6,9,10-heptachlorodecane (HeptaCD) to chicken and human liver microsomes. Two metabolites, monohydroxylated hexachlorodecane (HO-HexCD) and monohydroxy heptachlorodecane (HO-HeptaCD), were detected in human liver microsomal assays, while only one metabolite (HO-HexCD) was identified in chicken liver microsomal assays. After 1 h incubation, concentration of HeptaCD in chicken liver microsomes decreased by approximately 13%, but by 50% in human liver microsomes, indicating that the metabolic rate in human liver microsomes were higher than those in the chicken. The result of Lin et al. (2022) [115] confirmed that CP were extensively and rapidly metabolized after incubation with human microsomes. The number of metabolites in Lin et al. was lower than that found in He et al. (2021) [114], which could be due to the difference in experiment condition and the extraction method.

The metabolism of SCCPs 1-chlorodecane by cytochrome P450 enzymes was simulated to use a simplified model of active site of CYPs, a density functional theory. In this theory, 1-chlorodecane can be easily metabolized by CYPs in either low-spin or high-spin state, but hydroxylation was more major than dichlorination due to the energy barrier. The main metabolic products were 10-chloro-1-decanol, 10-chloro-decan-5-ol, and 1-chorodecanol with a rate constant of 42.3^−1^/h by CYPs in the human body [116].

## 6. Conclusions and Perspectives

In this article, the current studies on bioaccumulation and biotransformation of CPs in biota and humans were briefly reviewed. CPs including vSCCPs, SCCPs, MCCPs, and LCCPs are widely distributed in various biota, including humans, worldwide. The bioaccumulation and biotransformation of CPs are directly related to carbon chain and chlorination degree. However, our knowledge on the effects of chain length, chlorination degree, constitution, and stereochemistry on the bioaccumulation and biotransformation are still scarce. There are several issues relating to CPs, which should be considered research priorities:

There is an urgent need for relevant data on MCCPs and LCCPs. SCCPs have been regulated as POPs since 2017, while MCCPs and LCCPs remain unclassified (UNEP, 2017) [117]. Recent studies indicated that MCCPs, LCCPs, and vSCCPs were ubiquitous in the environment and they were also detected in biota samples. The concentrations of MCCPs and LCCPs were comparable or on the same order of magnitude with those of SCCPs. Some studies also reported the biomagnification or trophic magnification of MCCPs and LCCPs in aquatic and terrestrial food chains/webs. Considering the substantial amount of MCCPs and LCCPs produced compared to SCCPs, more concerns should be paid to MCCPs and LCCPs in further studies.

Future work is needed to study the bioaccumulation and influencing factors (such as the tissue distribution, degradation, and transformation) of CPs in aquatic and terrestrial food webs. Similar to other lipophilicity POPs, the lipid content of tissue plays a key role in the bioaccumulation and tissue distribution of CPs. However, the effects of the carbon chain length and chlorination degree on the bioaccumulation and tissue distribution are unclear. Inconsistent results on the biomagnification and trophic magnification of CPs were obtained according to available data. There is sufficient evidence to support the biotransformation of CPs by microorganisms, plants, and animals. However, knowledge gaps still exist. The knowledge on species-specific bioaccumulation and biotransformation is still scarce. Investigations on the biotransformation of CPs are still in the early stages. No field monitoring studies have been conducted to detect the metabolites of CPs. How and to what extent the species-specific biotransformations affect the bioaccumulation and trophic transfer of CPs are unknown.

Further investigation on the mechanisms of the toxicity of CPs are required. The bioaccumulative potential, toxicity, and biotransformation pathway vary for different CP congeners. Not all CPs are equally persistent towards biotransformation, and not all CP isomers are bioaccumulating and equally toxic. The effects of chain length, chlorination degree, constitution, and stereochemistry on the bioaccumulation, biotransformation, and the toxicity are unclear. Therefore, one can question whether a cut based on carbon chain length is a sufficient criterion for regulation or whether other criteria are needed to assess and classify medium- and long-chain CPs as well.

As CP metabolites are identified, the toxicity of CP metabolites needs to be evaluated and studied. Potential CP metabolite candidates, which may resemble chlorine-containing aliphatic molecule species, had adverse biological effects in earlier studies. Molecular biological methods can be used to conduct in-depth toxicological studies on CP metabolites at the molecular, gene, and cell levels.

It is necessary to explore the molecular mechanism of microbial degradation and possible phytoremediation of CPs among different biological species to determine the key process of the biological metabolism to reduce the occurrence of CPs in the environment.

## Figures and Tables

**Table 1:** Levels of CPs in the aquatic biota, terrestrial biota, humans, and human foodstuffs samples.

Site	Organisms	SCCPs		MCCPs		Reference
		Mean/Median	Range	Mean/Median	Range	
**Aquatic biota**						
Qingyuan, China	Fish and invertebrates		1700–95,000 ^a^			[13]
Qingyuan, China	Fish, snake, bird eggs		1200–250,000 ^a^		2300–200,000 ^a^	[17]
Qingyuan, China	Insects		52–410 ^c^		40–740 ^c^	[20]
Guiyu, China	Catfish	30,800 ^a^	11,400–70,400 ^a^			[16]
Hong Kong, China	Fishes	801 ± 253 ^a^	280–1940 ^a^	1820 ± 934 ^a^	502–4770 ^a^	[21]
	Crustaceans	422 ± 162 ^a^	202–694 ^a^	593 ± 306 ^a^	205–1190 ^a^	
	Mollusks	328 ± 79 ^a^	259–506 ^a^	603 ± 132 ^a^	464–874 ^a^	
Liao Estuary, China	Mollusks	66,500 ^a^	28,100–120,400 ^a^			[18]
Liaodong Bay, China	Fish	968 ^b^	53.3–2907 ^b^			[22]
Liaodong Bay, China	Invertebrates and fish	20,100 ^a^	2300–76,500 ^a^			[19]
Liaodong Bay, China	Fish		376.3–8596 ^a^		22.37–5097 ^a^	[23]
Bohai Sea, China	Bivalves	1710.5 ^b^	476.4–3269.5 ^b^			[24]
Pearl River Estuary, China	Marine organism		870–36,000 ^a^			[25]
Pearl River Estuary, China	Marine organism		210–21,000 ^a^			[26]
East China Sea	Marine organism	155 ± 215 ^c^	12.8–1819 ^c^			[27]
South China Sea	Finless porpoises	2800 ^a^	570–5800 ^a^	5100 ^a^	670–11,000 ^a^	[28]
	Humpback dolphins	5500 ^a^	920–24,000 ^a^	13,000 ^a^	1400–56,000 ^a^	
Bohai Sea, China	Mollusk	1410 ^b^	64.9–5510 ^b^			[8]
China	Farmed crabs	543 ^a^	82–1760 ^a^		nd–680 ^a^	[29]
Dianshan Lake, China	Wild aquatic organisms		10,000–1,300,000 ^a^			[30]
Taiwan, China	Freshwater Fish		nd–2,320,000 ^a^			[31]
Hainan, China	Coral	/800 ^a^	184–7410 ^a^	/1490 ^a^	305–14,800 ^a^	[32]
China	Fish of the Pearl Real Delta	16,000 ± 12,000 ^a^	3000–41,000 ^a^			[33]
	Farmed freshwater fish	5900 ± 8100 ^a^	220–51,000 ^a^			
	Fish of the Yangtze River Delta	3000 ± 1600 ^a^	900–7300 ^a^			
Baltic Sea	Marine organisms		nd–220 ^a^		nd–390 ^a^	[34]
Antarctic	Fish and mosses	262 ^b^	69.0–504 ^b^			[22]
Antarctic	Fish		1500 ± 500 ^a^			[35]
Ny-Ålesund and London Island, Svalbard, Arctic	Algae, gammarids, and cod	178.9 ^b^				[36]
Persian Gulf, Iranian	Larak coral tissue	112 ^b^	28.5–200 ^b^	65.9 ^b^	15.5–136 ^b^	[37]
France	Fish	/728 ^a^	63–1492 ^a^	/4430 ^a^	99–11,300 ^a^	[38]
Antarctic	Humpback whales		nd–46 ^a^			[39]
Northern Europe	Fish and seabird		28–880 ^a^		14–3700 ^a^	[40]
North Baltic Sea	Liver of fish		19–286 ^c^		25–260 ^c^	[41]
Greenland and Iceland and Sweden	Bivalves marine mammals		<5.2–570 ^a^		<8.6–270 ^a^	[42]
Norway	Herring gull blood and eggs		5–200 ^c^		3–630 ^c^	[43]
German	Aquatic and terrestrial organisms		nd–350 ^a^		nd–1800 ^a^	[44]
Greenland, Denmark	Bird and marine mammals		220–2200 ^c^			[10]
South Korea	Black-tailed gull eggs		1180–2931 ^a^		694–2023 ^a^	[45]
**Terrestrial biota**						
Taizhou, China	Apple snail	314 ^b^	137–821 ^b^			[46]
Guiyu, China	Chicken eggs		2300–6800 ^a^			[47]
	Goose eggs		nd–150,000 ^a^			
Guiyu, China	Pigeon	7600 ^a^	4700–11,000 ^a^			[16]
Qingyuan, China	Bird species		620–17,000 ^a^			[48]
Qingyuan, China	Chicken		460–13,000 ^a^			[49]
Qingyuan, China	Insect		2200–9000 ^a^		990–11,000 ^a^	[50]
	Birds		6100–48,000 ^a^		2000–33,000 ^a^	
	Frogs and toads		8100–24,000 ^a^		4600–17,000 ^a^	
Tibetan, China	tree bark		2900–7000 ^a^		1800–5700 ^a^	[51]
	needle		2400–6400 ^a^		1600–5000 ^a^	
	lichen		1400–6100 ^a^		700–4000 ^a^	
	moss samples		1500–5300 ^a^		900–4000 ^a^	
Tibetan, China	Plants	4300 ± 2830 ^a^				[14]
	Plateau pika	1870 ± 1090 ^a^				
	Eagle	723 ± 536 ^a^				
Tibetan, China	Chicken and goose eggs		3098–6999 ^a^			[52]
China	Mature maize plant	381 ^b^	119–61,999 ^b^	551 ^b^	77.6–52,930 ^b^	[53]
Beijing, China	Plants	127 ± 116 ^b^	13–593 ^b^	289 ± 148 ^b^	21–785 ^b^	[54]
German	Fauna sample (mussels, fish, birds, earthworms, and roe deer) Flora sample (tree shoots and leaves)		nd–350 ^a^		nd–1800 ^a^	[44]
Greenland, Denmark	Black guillemot eggs Glaucous gull liver Ringed seal blubber Polar bear adipose tissue		220–2200 ^c^			[10]
South Korea	Black-tailed gull (Larus crassirostris) eggs		1180–2931 ^a^		694–2023 ^a^	[45]
**Humans and human foodstuffs**						
Beijing, China	Maternal serum		21.7–373 ^c^		3.76–31.8 ^c^	[55]
	Cord serum		8.51–107 ^c^		1.33–12.9 ^c^	
China	Breast milk in urban and rural areas	/393 ^a^ /525 ^a^	131–808 ^a^ 139–1543 ^a^	/472 ^a^ /576 ^a^	94–1714 ^a^ 211–1089 ^a^	[56]
Henan, China	Human placentas	593 ^a^	98.5–3771 ^a^	316 ^a^	80.8–954 ^a^	[57]
China	Hair	/239 ^b^	19.2–877 ^b^	/325 ^b^	16.9–893 ^b^	[58]
	Nails	/154 ^b^	57.7–355 ^b^	/233 ^b^	61.0–476 ^b^	
China	Cereal samples	343 ^c^		51.6–981 ^c^	213 ^c^	[59]
	Legume samples	328 ^c^		47.1–801 ^c^	184 ^c^	
Jinan, China	Human serum	/13,800 ^a^	1670–42,700 ^a^	/15,200 ^a^	1350–38,900 ^a^	[60]
China	Human blood	/3500 ^a^	370–35,000 ^a^	/740 ^a^	130–3200 ^a^	[61]
China	Maternal serum,	/117,100 ^d^		/38,900 ^d^		[62]
	Cord serum,	/70,000 ^d^		/25,600 ^d^		
	Placenta	/30.3 ^c^		/19.0 ^c^		
	Breast milk	/82,600 ^d^		/26,100 ^d^		
China	Human milk	/303 ^a^		/35.7 ^a^		[63]
China, Korea, Japan	Breast milk		nd–54 ^a^ nd–20 ^a^ nd–20 ^a^			[64]
South China	Maternal		3280–10,400 ^d^		1300–5500 ^d^	[65]
	cord blood		890–4130 ^d^		890–1690 ^d^	
	placenta		3.18–9.12 ^c^		1.91–4.89 ^c^	
Taizhou, China	Paddy seeds	17.6 ^b^	4.9–55.1 ^b^			[46]
Jinan, China	Food samples	69.3 ± 74.4 ^c^	8.27–268 ^c^			[66]
China	Aquatic food samples	1472 ^c^	215–4200 ^c^	80.5 ^c^	9.0–586 ^c^	[67]
China	Cooking oil		<9–7500 ^c^			[68]
China	Cooking oil		nd–16,055 ^c^		nd–11,612 ^c^	[69]
China	Meat	129 ± 4.1 ^c^	15.7–469 ^c^	5.7 ± 0.59 ^c^	0.3–23.8 ^c^	[70]
China	Infant formulas,	7.95 ^c^	2.32–54.2 ^c^		1.67–20.9 ^c^	[71]
	Cereals	4.26 ^c^	2.73–8.81 ^c^		1.21–8.24 ^c^	
	Purees	4.66 ^c^	1.33–8.43 ^c^		0.53–5.41 ^c^	
China	Food samples		nd–120 ^c^		nd–100 ^c^	[72]
China	Green tea	55.7 ^b^	4.99–717 ^b^	33.5 ^b^	2.55–543 ^b^	[73]
China	Noodle	1200 ^c^	59–3000 ^c^	140 ^c^	12–520 ^c^	[74]
	Seasoning	1400 ^c^	160–3300 ^c^	160 ^c^	8–650 ^c^	
	Noodle soup	560 ^d^	160–1500 ^d^	540 ^d^	19–1500 ^d^	
China	Raw milk	1470 ^a^	130–5770 ^a^	170 ^a^	6.8–800 ^a^	[75]
German	Infant food		nd–190 ^a^		Nd–32 ^a^	[76]
Europe	Baby food		nd–3765 ^a^			[77]
German	Vitamin E supplements	3810 ^a^	nd–61,100 ^a^	15,200 ^a^	nd–151,000 ^a^	[78]
UK	Human milk	/180 ^a^	49 to 820 ^a^	/21 ^a^	6.2 to 320 ^a^	[79]
Czech	Human serum	/370 ^a^	150–2600 ^a^	/360 ^a^	200–2110 ^a^	[80]

^a^: Unit is ng/g lw, ^b^: ng/g dw, ^c^: ng/g ww, ^d^: ng/L, nd: no detect. /data: indicated median.

## Data Availability

No applicable.
